# Classifying synoptic patterns driving tornadic storms and associated spatial trends in the United States

**DOI:** 10.1038/s41612-025-00897-1

**Published:** 2025-01-08

**Authors:** Qin Jiang, Daniel T. Dawson II, Funing Li, Daniel R. Chavas

**Affiliations:** 1 Department of Earth, Atmospheric, and Planetary Sciences, Purdue University, West Lafayette, IN USA; 2 Department of Earth, Atmospheric, and Planetary Sciences, Massachusetts Institute of Technology, Cambridge, MA USA

**Keywords:** Climate sciences, Atmospheric science, Atmospheric dynamics

## Abstract

Severe convective storms and tornadoes rank among nature’s most hazardous phenomena, inflicting significant property damage and casualties. Near-surface weather conditions are closely governed by large-scale synoptic patterns. It is crucial to delve into the involved multiscale associations to understand tornado potential in response to climate change. Using clustering analysis, this study unveils that leading synoptic patterns driving tornadic storms and associated spatial trends are distinguishable across geographic regions in the U.S. Synoptic patterns with intense forcing featured by intense upper-level eddy kinetic energy and a dense distribution of Z500 fields dominate the increasing trend in tornado frequency in the southeast U.S., generating more tornadoes per event. Conversely, the decreasing trend noted in certain regions of the central Great Plains is associated with weak upper-level synoptic forcing. These findings offer an explanation of observational changes in tornado occurrences, suggesting that the physical mechanisms driving those changes differ across regions.

## Introduction

Severe convective storms and tornadoes rank among nature’s most hazardous phenomena, inflicting significant property damage and casualties^1–3^. Tornadoes account for nearly one-fifth of all natural hazard fatalities in the United States and have caused 1775 fatalities and 25,959 casualties over the 24-year period 1995–2018^4^. The annual economic damages range from *$*183 million to *$*9.5 billion, and a single event can approach up to *$*3 billion^5^. Understanding tornado potential is critical for mitigating these associated risks.

**Fig. 1:**
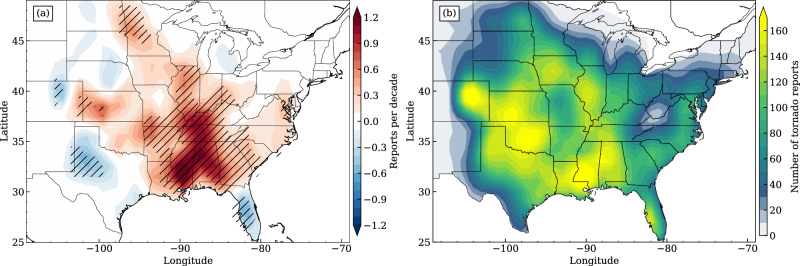
Spatial trends and distributions of tornado reports in the U.S. **a** The distribution of Theil-Sen slopes of 1980–2022 annual gridded tornado reports. Hatched areas denote the trends significantly differ from 0, assessed at *p* values ≤0.05 using Kendall’s *τ* statistic. **b** The number of tornado reports per 1° × 1° cells from 1980 to 2022.

How tornado potential will change in response to the present warming climate remains elusive but has been an intriguing question of high societal and research interest. Increased variability of tornado frequency has been reported due to a greater concentration of tornadoes on fewer tornado days^6–9^, including more tornadoes in the most extreme tornado outbreaks^10,11^. Recent research has suggested spatial trends in tornado frequency in the United States based on historical tornado reports^12–14^. Notably, negative tornado frequency trends have been observed in certain Great Plains regions, while the Southeast United States shows positive trends (Fig. 1a; extended to 2022 following the method in ref. ^13^). Considering the uncertainty in observational reports^15^, many studies have used environmental proxies to approximate tornado favorability in reanalysis and/or rawinsondes^11,13,16–21^, and global climate models^22,23^. Despite these findings, relatively little is known of the physical mechanisms driving those trends, probably owing to the low predictability of tornadic storms^24,25^, which are sensitive to both large-scale synoptic patterns and finer-scale local characteristics.

The regions east of the Rocky Mountains in the U.S. are known for their high frequency of tornadoes (Fig. 1b). The prevailing conceptual model for the generation of storm environments in those regions emphasizes the key role of the elevated terrain of the Rocky Mountains^26–30^ and upstream surface conditions^31^. Meanwhile, a typical synoptic pattern during a tornado outbreak usually involves an upper-level trough axis west of the location of tornadogenesis. The associated rapid surface pressure falls intensify the low-level jet and associated moisture advection, favoring storm potential^32,33^. Variabilities in atmospheric circulation patterns associated with Arctic sea-ice extent^34^, Madden-Julian oscillation (MJO) modes^35^, and El Niño/Southern Oscillation (ENSO) phases^36,37^, along with other teleconnections^38^ have been found to affect the storm’s environmental background, altering tornado potential across different U.S. regions. Studies using regime classification also show that the location, frequency, and predictability of U.S. tornadoes are modulated by different weather pattern categories^37,39^.

Based on these findings, our outstanding research question is: do different synoptic patterns contribute differently to the spatial trends of tornado frequency across geographic regions in the U.S.? Given that tornadoes are spread widely over regions east of the Rocky Mountains (Fig. 1b), including cold season tornadoes^40^ and those beyond traditional “tornado alley”^41^, a related question also includes how leading synoptic patterns driving tornadic storms may vary by regions and/or seasons.

This study employs hierarchical clustering analysis to classify the leading synoptic patterns driving tornadic storms across different geographic regions east of the Rocky Mountains in the U.S., based on historical tornado reports and reanalysis datasets. The classification is based on tornado-relative synoptic backgrounds and hence is not dependent on pre-determined geographic or temporal constraints. How these different synoptic patterns establish storm-favorable environments and, more importantly, how they contribute to the spatial trends of tornado reports will be of main interest. We found that the leading synoptic patterns across regions are distinguishable, and the increasing trend in tornado frequency in the southeastern U.S. is mainly driven by synoptic patterns with intense forcing, while the decreasing trends in portions of the Great Plains are associated with weaker synoptic forcing. These findings suggest that the physical mechanisms driving the spatial trends of tornado occurrences differ across regions in the U.S.

## Results

### Leading synoptic patterns driving tornadoes across geographic regions

Previous attempts to detect storm-favorable synoptic patterns have mainly focused on tornado outbreaks^32,33,42^, but they are only a small portion of the tornado reports. We employ a hierarchical cluster analysis of all tornado reports east of the Rocky Mountains in the U.S., excluding those from Florida, to track the highly generalized tornado-occurring synoptic patterns. We first create a dataset of meteorological fields relative to the report start position and apply 500 hPa height (Z500) fields to the clustering algorithm to characterize synoptic features. The number of final clusters is defined based on an abrupt increase in a measure of dissimilarity (See Methods and Supplementary Fig. S1), and field variables within each final cluster are then averaged to provide composite synoptic patterns and storm environments.

**Fig. 2:**
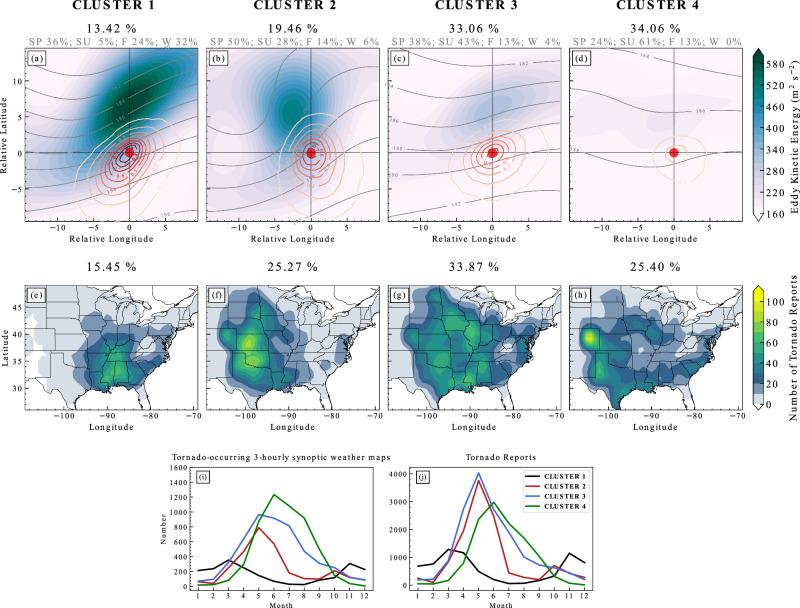
Predominant synoptic patterns driving tornadoes across regions. **a**–**d** The tornado-relative distribution of composite eddy kinetic energy (EKE) at 200 hPa (color fills; m^2^ s^−2^), normalized Z500 multiplied by 10^3^ (grey contours; unitless), significant tornado parameter (red contours; unitless) within each cluster, respectively. The red dots denote the tornado report start location, and the *x*- and *y*-axes denote the tornado-relative longitude and latitude. The black percentage values on the title indicate the percentage of the number of tornado-occurring 3-hourly synoptic weather maps included in each cluster, and the grey subtitle shows the relative percentage for each season (SP: Spring; SU: Summer; F: Fall; W: Winter) within each cluster. **e**–**h** The spatial distribution of tornado reports associated with each synoptic cluster. The percentage values indicate the percentage of the number of tornado reports within each cluster. **i**, **j** Monthly variation of counts of tornado-occurring 3-hourly synoptic weather maps and tornado reports for each cluster, respectively.

Figure 2 shows the composite synoptic fields of the final four clusters. In **Cluster 1**, the intense upper-level jet streak described by the high values of eddy kinetic energy (EKE) associated with a dense distribution of Z500 contours (Fig. 2a) dominate the tornado events in the southeast U.S. (Fig. 2e). These synoptic conditions prevail in the cold season (November-March; shown in the black curve in Fig. 2i, j). **Cluster 2** describes a deeper trough to the west of the tornadogenesis position (Fig. 2b) but with weaker EKE aloft than **Cluster 1**. **Cluster 2** is mostly comprised of tornadoes in the central U.S. in late Spring and early Summer (the red curve in Fig. 2i and j). Meanwhile, **Cluster 3** reveals weaker EKE and a shallower trough feature (Fig. 2c), with its associated tornadoes (roughly 1/3 of our subset) lacking a clear geographic hotspot (Fig. 2g) and occurring broadly in the warm season (the blue curve in Fig. 2j). **Cluster 4** is associated with minor synoptic forcing aloft (Fig. 2d), which is commonly seen in Summer (the green curve in Fig. 2i and j), associated with tornadoes closer to the lee side of the Rocky Mountains (Fig. 2h).

Given that we perform the hierarchical clustering based on the tornado-relative Z500 fields, the spatial information is removed. However, it turns out that the distinct distribution of the associated tornado reports is caused by spatial differences in leading synoptic patterns. As seen in Supplementary Fig. S2, synoptic patterns in **Cluster 1** prevail in the eastern U.S., **Cluster 2** dominates the central U.S., and **Cluster 4** is closer to the climatological mean.

### Synoptic processes governing tornado-favorable storm environments

**Fig. 3:**
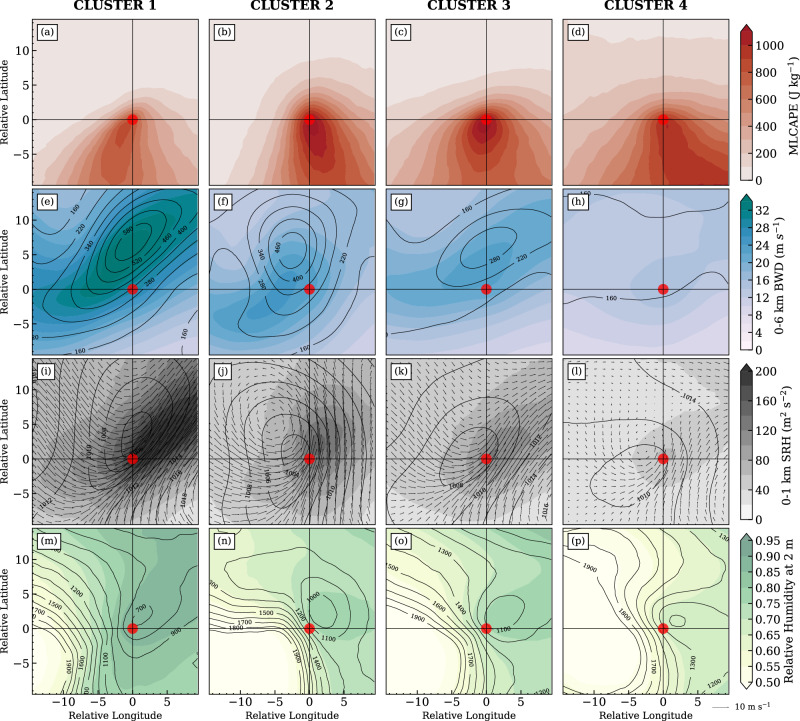
Storm environmental conditions associated with each synoptic pattern. Composite distributions of environmental parameters within each cluster (each column), respectively: **a**–**d** mixed-layer-based CAPE (MLCAPE; J kg^−1^); **e**–**h** 0–6 km bulk wind difference (BWD06; color fills; m s^−1^) and eddy kinetic energy at 200 hPa (EKE; black contours; in 60-m^2^ s^−2^ intervals); **i**–**l** 0-1 km storm-relative helicity (SRH01; color fills; m^2^ s^−2^), sea-level pressure (black contours; in 2-hPa intervals) and wind vector at 850 hPa. **m**–**p** Relative humidity at 2 m AGL (color fills) and mixed-layer-based lifting condensation level (MLLCL; black contours; in 100-m intervals). The red dots denote the tornado report start location, and the x- and *y* axes denote the tornado-relative longitude and latitude.

It is well documented that tornadoes occur in environments characteristic of high values of thermal instability, adequate surface to mid-level vertical wind shear, abundant near-surface moisture supply, and strong storm-relative helicity in the lowest 1 km of the boundary layer^43–45^. These storm environments are commonly described by mixed-layer-based convective available potential energy (MLCAPE), 0–6 km bulk wind difference (BWD06), and 0–1 km storm-relative helicity (SRH01) (see Methods). It’s known that the stretching of potential vorticity associated with the upstream side of a trough enhances the surface pressure falls, intensifying the low-level jet^46^. In addition, the development of low-level jets is also coupled with upper-level jet streaks as the return branches of transverse circulations, which are forced by mass and momentum adjustments^47^. The development of convective systems can also amplify the large-scale flow pattern, promoting storm-favorable conditions^48^. These multiscale jet feedbacks are particularly important for strongly forced synoptic regimes^48^. Therefore, the low-level meteorological features are highly coupled with upper-level atmospheric characteristics. As seen in Fig. 3, the more intense synoptic forcing, decreasing from **Cluster 1** to **Cluster 4**, is associated with higher upper-level EKE (comparing Fig. 3e–h) and lower low-level pressure (comparing Fig. 3i–l), which intensifies the low-level jet and associated moisture transport, resulting in a lower mixed-layer-based lifting condensation level (MLLCL; comparing Fig. 3m–p). Together, these create conditions favorable for tornadogenesis. The characteristics of the upper-level EKE also control the strength and distribution of both BWD06 (Fig. 3e–h) and SRH01 (Fig. 3i–l), generating a vertically tilted structure with low-level SRH01 placed to the southeast of the upper-level EKE (comparing the position of EKE maxima in Fig. 3e–h with SRH01 maxima in Fig. 3i–l). **Cluster 1** shows a lower-CAPE (Fig. 3a) higher-shear (Fig. 3e) storm environment than others, characterizing cold season storm conditions^49^ (shown as the black curve Fig. 2i). The **Clusters 2** and **3** illustrate higher-CAPE (Fig. 3b and c) but lower-shear (Fig. 3f, g) conditions than **Cluster 1**, but the shear values still fall within the bound of typical high-shear storm environments (≥18 m^−1^^43,49^). In association with minor upper-level synoptic forcing, **Cluster 4** exhibits low-shear (Fig. 3h) and low-SRH (Fig. 3l) conditions but toward the upper-end of “low” parameter space, corresponding to typical summer storm patterns (shown as the green curve Fig. 2i), which could lead to more isolated convective storms. The reader shall also note that near-storm environments still show large variabilities on a case-by-case basis. For example, a tornadic storm may be affected by mesoscale characteristics and develop outside the ideal location governed by synoptic forcing. The kernel density distribution of storm environments that quantifies the variations in cases is shown in Supplementary Fig. S3.

### Synoptic patterns contributing to spatial trends in tornado frequency

**Fig. 4:**
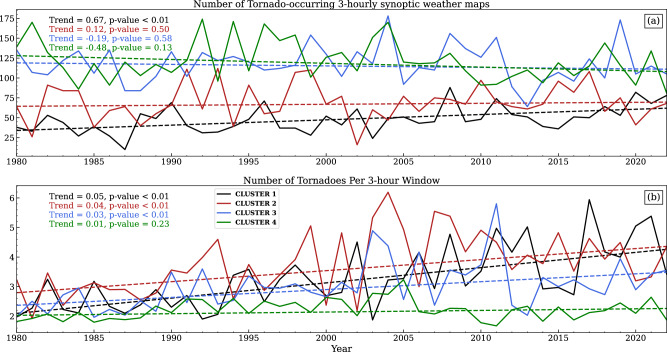
Trends in the frequency of synoptic patterns and associated tornado reports. Interannual changes of **a** the annual counts of tornado-occurring 3-hourly synoptic weather maps (solid lines) and **b** the number of tornadoes per 3-hour window (solid lines; calculated by dividing the annual number of tornadoes by the annual number of tornado-occurring weather maps) for each cluster, respectively. The dashed lines indicate the linear regression predictor for each series. The trend values denote the slope of the linear regression using the Theil-Sen estimator. The *p* values are achieved using Kendall’s *τ* statistics, and a value ≤0.05 indicates a statistically significant trend that differs from 0 (no change).

Based on the different leading synoptic patterns recognized, this section seeks to explore how each pattern may contribute to the spatial trends in tornado frequency in different ways. As shown in Fig. 4, **Cluster 1** is the only one that shows significant increasing trends in both the annual number of tornado-occurring 3-hourly synoptic weather maps and annual mean tornado frequency within 3-hour windows (calculated by dividing the annual number of tornadoes by the number of tornado-occurring weather maps). Although not statistically significant, **Cluster 3** and **4** show a decreasing trend in tornado-occurring 3-hour weather maps (Fig. 4a), and those are primarily associated with weaker synoptic forcing as shown in Figs. 2 and 3. **Clusters 1–3** show a significant increase in tornado frequency per 3 hours, and a larger trend value is associated with the cluster with more intense synoptic forcing (Fig. 4b). Those indicate that synoptic patterns with intense forcing (**Cluster 1**) become more frequent in the past 43 years of the warming scenario, and each synoptic event has triggered more tornadoes associated with it.

**Fig. 5:**
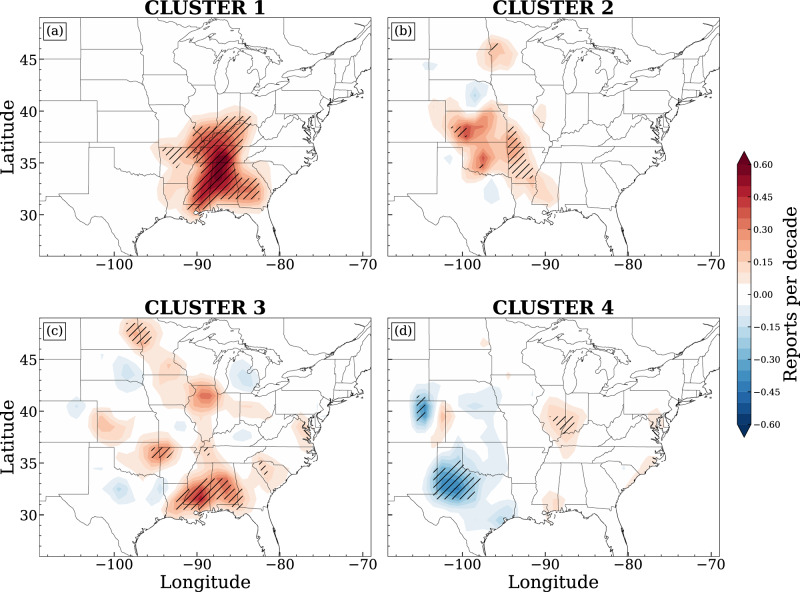
The spatial distribution of Theil-Sen slopes of gridded annual tornado reports for each cluster, respectively. Areas with trends significantly different from 0 (no change) are hatched, assessed at *p* values ≤0.05 using Kendall’s *τ* statistic.

**Fig. 6:**
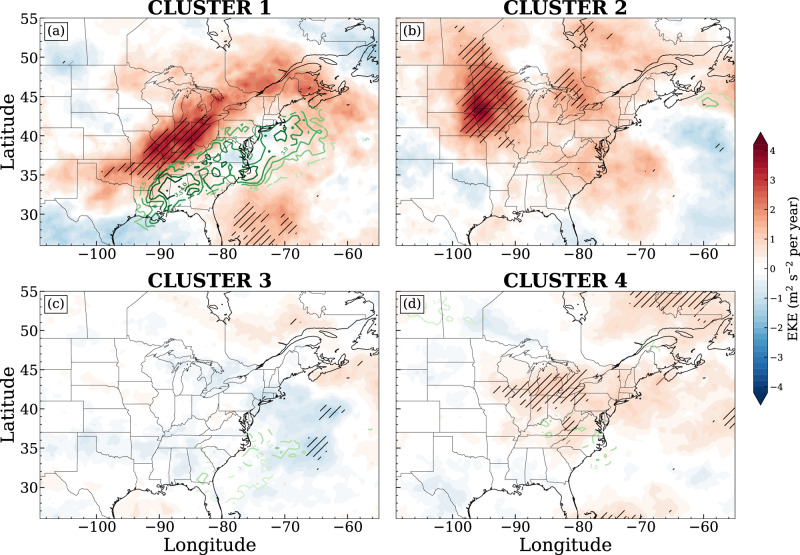
The distribution of Theil-Sen slopes of annual median eddy kinetic energy (EKE; diverging color fills; m^2^ s^−2^ per year) for each cluster, respectively. Areas with trends significantly different from 0 (no change) are hatched, assessed at *p* values ≤0.05 using Kendall’s *τ* statistic. Green contours denote trends in annual median integrated vapor transport (IVT; kg m^−1^ s^−1^ per year; magnified by 10^2^), with only areas with significant trends displayed for clarity.

The detailed spatial distribution of trends in tornado frequency is seen in Fig. 5. **Cluster 1** dominates the primary increasing trend in tornadoes in the southeast U.S, with no decreasing patterns observed in Fig. 5a. While **Cluster 2** and **4** both govern tornadogenesis in the central U.S., their contributions to the tornadoes’ spatial trends are distinct from each other. **Cluster 2** still mainly contributes to the increasing trend in the central U.S. (Fig. 5b), although the slope is much smaller than **Cluster 1**. Conversely, **Cluster 4** dominates the decreasing trend in the central U.S., especially in Texas and eastern Colorado (Fig. 5d). **Cluster 3** highlights uncertainties that cannot be attributed to the other patterns (Fig. 5c). In combination with the analysis of leading synoptic patterns (Fig. 2), these analyses also indicate that the spatial trends in tornado frequency response to synoptic forcing are different. Notably, the increasing trend in tornado frequency is mainly driven by intense synoptic forcing. These tornado-associated intense synoptic patterns are also themselves intensified, as seen in the robust increases in upper-level annual median EKE (Fig. 6a; diverging color fills), which is in turn highly associated with the enhanced water vapor transport to the southeast (Fig. 6a; green contours). This suggests that the multiscale interaction between jets, moisture supply, and storm behaviors plays a key role in the increased tornado risk in the southeastern U.S., warranting further investigation. Meanwhile, the decreasing trend is associated with weaker synoptic forcing and with no robust association with upper-level jet characteristics (Fig. 6d).

### Uncertainties and robustness

Due to inherent statistical uncertainties^50^ and significant variability in storm environments compared to generalized leading synoptic patterns, different clustering methods and sample inputs may influence how certain tornado-associated weather maps are categorized within each cluster. Despite categorization biases, such as differences in percentage values [cf. Fig. 2 with Supplementary Fig. S4 using K-Mean and Supplementary Fig. S5 using Self-Organization Maps (SOM)], the overall characteristics of the resulting four synoptic patterns do not substantially differ when different clustering methods are used. Another limitation of our analysis is a dependence on the report-based dataset, which has recognized issues and biases^15,51^, especially regarding EF0 and EF1 tornado reports. However, previous studies using environmental proxies^13^ also support the observed spatial trends in tornado reports. Tests confirm that randomly sampling EF0/EF1 reports (Supplementary Figs. S6, S9, and S12) or excluding EF0 reports (Supplementary Figs. S7, S10, and S13) do not alter our key findings. Moreover, additional processing of the synoptic dataset, such as detrending the Z500 field^37,52^, may cause the aforementioned sampling variability, but the resulting four synoptic patterns remain consistent as normalization is always performed prior to clustering. We use MERRA-2 in our main manuscript, but the results using the European Centre for MediumRange Weather Forecasts Reanalysis version 5 (ERA5) dataset with its original resolution (no interpolation is performed) again are not appreciably different (shown in Supplementary Figs. S8, S11, and S14), confirming that our main conclusions remain robust.

## Discussion

Understanding the mechanisms driving tornadic storms and their changes in a warming climate is essential for mitigating future risks. This study reveals that the leading synoptic patterns driving tornadic storms and associated spatial trends are distinguishable across geographic regions in the U.S. The intense upper-level jet streak described by high values of EKE associated with the dense distribution of Z500 contours dominates the tornado events in the southeast U.S. in the cold season (November-March). Late Spring and early Summer tornado events in the central and southern Great Plains are dominated by deep trough systems to the west of the tornadogenesis position, while more summer events associated with weak synoptic forcing are positioned closer to the lee side of Rocky Mountains. These distinct synoptic patterns also govern the trends of tornado frequency over the past 43 years in different regions of the U.S. Specifically, the increasing trend in tornado frequency in the southeast U.S. is mainly driven by synoptic patterns with intense forcing, and the intense synoptic patterns are also become more frequent, driving more tornadoes per synoptic event. The decreasing trend in certain regions of the Great Plain is associated with weaker synoptic forcing.

Our findings partially explain the observational changes in tornado occurrence in the U.S. over the past decades of the warming scenario, referring to fewer tornado days but more tornadoes per tornado day, along with spatial trends indicating more tornadoes in the southeast regions. However, diagnosing the mechanisms contributing to these synoptic-scale trends may be more difficult. On the one hand, the synoptic patterns with intense forcing in **Cluster 1** and **2** also show a robust increase in upper-level EKE (Fig. 6). This trend is expected to be correlated with a change in upper-level jet characteristics and global circulation. For example, fast upper-level jet stream winds have been proposed to accelerate more than the average under climate change following the “moist-get-moister” response that affects the thermal wind^53^. A poleward shift of the mid-latitude jet stream could contribute to more frequent atmospheric rivers in the eastern U.S.^54^ Alternatively, the decreasing trends are more likely to be affected by local features. For instance, a change in boundary layer characteristics may dominate the decreasing trend in certain regions of the central U.S. as the upper-level synoptic forcings are weak. Those may involve a larger value of CIN^23,55^ and reduced soil moisture^56,57^ that inhibit the buildup of thermal energetics^58,59^. The surface processes associated with various land cover types across regions may become more important in modulating storm behavior^31,60–62^ in this scenario. This study emphasizes the need for collaborative projects that incorporate expertise from both climate and mesoscale scientists to conduct multiscale investigations to explore the underlying physics in more depth.

## Methods

### Datasets

The tornado data were retrieved from the Storm Prediction Center (SPC)’s online database (https://www.spc.noaa.gov/wcm/#data) for the period of 1980–2022. As tornadoes are most frequent east of the Rocky Mountains, we focused on tornado activities east of the 115° longitudinal boundary. We intentionally omitted reports in Florida before applying clustering analysis because the storm environments in Florida may primarily feature tropical atmospheric conditions, which differ from other typical synoptic conditions in the U.S. Therefore, the tornado reports in Florida were not shown in results except for Fig. 1. To yield the spatial distribution (Fig. 1b), tornado reports were first binned on a 1° × 1° grid and then smoothed in space using a 1-*σ* Gaussian kernel. The meteorological environments were based on the National Aeronautics and Space Administration’s (NASA) Modern-Era Retrospective analysis for Research and Applications Version 2 (MERRA-2^63,64^) 3-hourly surface and pressure-level reanalysis data with a horizontal resolution of 0.625° × 0.5°. The 3-hourly reanalysis data closest to the actual time of the tornado reports were chosen to represent the tornado-occurring synoptic conditions, and those were then truncated into a 30° × 30° spatial region surrounding the report start location (20° to the north or west and 10° to the south or east) to generate tornado-relative weather maps. Therefore, each weather map was associated with one or more tornado reports. We simply used the longitude and latitude mean as the genesis point if multiple reports were detected in the same 3-hour window. All told, 46240 tornado reports were used associated with 15313 tornado-relative weather maps.

### Clustering of synoptic patterns

We then performed the classification of synoptic patterns based on the 500 hPa height (Z500) from tornado-relative weather maps. Each tornado-relative Z500 field was first normalized by L2 normalization; in this way, the key differences featured by the clustering method mainly came from the pattern distribution instead of the matrix norm. The hierarchical clustering algorithm used in this study is an agglomerative approach (bottom-up), which merges pairs of similar clusters through consecutive steps until a single cluster is obtained at the end. The initial cluster dissimilarity was specified by the Euclidean distance, and the dissimilarity after merging was recursively computed via Ward’s minimum variance method^65^. The number of output clusters that represent distinct synoptic patterns was then determined by an abrupt increase in Ward’s distance (Supplementary Fig. S1), resulting in four final clusters in our studies. Field variables within each final cluster were then averaged to provide composite synoptic patterns or storm environments. The main advantage of hierarchical clustering over other methods, such as SOM^66,67^ or K-means clustering^68^, is that it does not require the users to prespecify the size of output clusters.

### Environmental parameters

The Significant tornado parameter (STP) is a composite of several environmental parameters^44,45,69^. The formulation was based on mixed-layer-based parcels:

1 $$\begin{array}{ll}STP=\frac{MLCAPE}{1500\,J\,k{g}^{-1}}\times \frac{2000-MLLCL}{1000\,m}\times \frac{200+MLCIN}{150\,J\,k{g}^{-1}}\\\qquad\quad\times \frac{SRH01}{150\,{m}^{2}\,{s}^{-2}}\times \frac{BWD06}{20\,m\,{s}^{-1}}\end{array}$$

using mixed-layer-based convective available potential energy (MLCAPE), mixed-layer-based lifting condensation level (MLLCL), 0–1 km storm-relative helicity (SRH01), 0–6 km bulk wind difference (BWD06). MLCAPE, MLCIN, and SRH01 were calculated based on retrieved soundings from MERRA-2 with their original definitions. For example, MLCAPE was derived from the vertical integration of buoyancy based on a mixed-layer lifted parcel from the level of free convection (LFC) to the equilibrium level after virtual temperature correction. MLCIN was calculated from the integration of negative buoyancy below the LFC, and 0–1 km SRH was obtained through the integration of storm-relative streamwise vorticity within that height range. We used the XCAPE Python package^70^, which had been used in many previous studies^18,23,31,71,72^, to perform those calculations with their default settings, including the specification of the lowest 500 m as the mixed layer. The mixed-layer lifted parcel has been suggested to be more representative of the actual parcel contributing to the convective cloud when computing these thermodynamic parameters^73^. Following the approach of refs. ^44,45^ in STP calculation, the MLLCL term was set to 1.0 for MLLCL <1000 m and set to 0.0 for MLLCL >2000 m; the MLCIN term was set to 1.0 for MLCIN > −50 J kg^−1^, and set to 0.0 for MLCIN < −200 J kg^−1^; the BWD06 term was set to 1.5 for BWD06 > 30 m s^−1^, and set to 0.0 when BWD06 < 12.5 m s^−1^.

The integrated water vapor transport (IVT) was calculated based on the equation^74,75^:

2 $$IVT=-\frac{1}{g}\int_{1000}^{200}q(p)| {{{\bf{V}}}}_{h}(p)| dp$$

where *q* denotes specific humidity (kg kg^−1^) in pressure levels, **V**_*h*_ = [u, v] denotes the horizontal wind vector (m s^−1^), and *p* denotes pressure levels.

### Trends

We performed the Theil-Sen estimator^76,77^ to detect the interannual linear trends of tornado reports. This method chooses the median of the slopes of all lines through pairs of input points and, hence, is insensitive to outliers and efficient in computation. Therefore, omitting any year of data, such as the super tornado outbreak in April 2011, has minimal impact on the results. Kendall’s *τ* statistic^78^ and a *p* value of 0.05 was used to examine the significance of the Theil-Sen slope. Both Theil-Sen Slope and *p* values were calculated at each grid point on the annual sum of reports.

## Supplementary information

Supplementary Information

## Data Availability

The tornado reports were retrieved from the SPC's online database (https://www.spc.noaa.gov/wcm/#data) for the period of 1980–2022. The surface and pressure-level MERRA-2 reanalysis data during the same period were downloaded from https://disc.gsfc.nasa.gov/datasets/M2I1NXASM_5.12.4/summary and https://disc.gsfc.nasa.gov/datasets/M2I3NPASM_5.12.4/summary. ERA5 reanalysis data are available at https://cds.climate.copernicus.eu/datasets/reanalysis-era5-pressure-levels.

## Code availability

Scipy package was used to perform hierarchical clustering analysis https://docs.scipy.org/doc//scipy-1.2.3/reference/cluster.hierarchy.html and Theil-Sen estimator https://docs.scipy.org/doc/scipy/reference/generated/scipy.stats.theilslopes.html. The storm environmental parameters were computed based on XCAPE Python package 10.5281/zenodo.5270332. Sklearn package was used to perform K-Mean https://scikit-learn.org/stable/modules/generated/sklearn.cluster.KMeans.html and Self-organization Map https://sklearn-som.readthedocs.io/en/latest/ clustering analysis in Supplementary materials. All scripts are available upon request.
