# Supplementary material for: Classifying synoptic patterns driving tornadic storms and associated spatial trends in the United States
Source: NPJ Clim Atmos Sci. 2025 Jan 8;8(1):7. doi: 10.1038/s41612-025-00897-1 (PMC11711087; doi:10.1038/s41612-025-00897-1)
Supplement: Supplementary file 1 — Supplementary Information [file 41612_2025_897_MOESM1_ESM.pdf]

Supporting Information for

Classifying synoptic patterns driving tornadic storms and associated spatial trends in the United States

Qin Jiang, Daniel T. Dawson II, Funing Li, and Daniel R. Chavas

Corresponding Author: Qin Jiang
drqinjiang@gmail.com

This PDF file includes:

Figs. S1 to S14

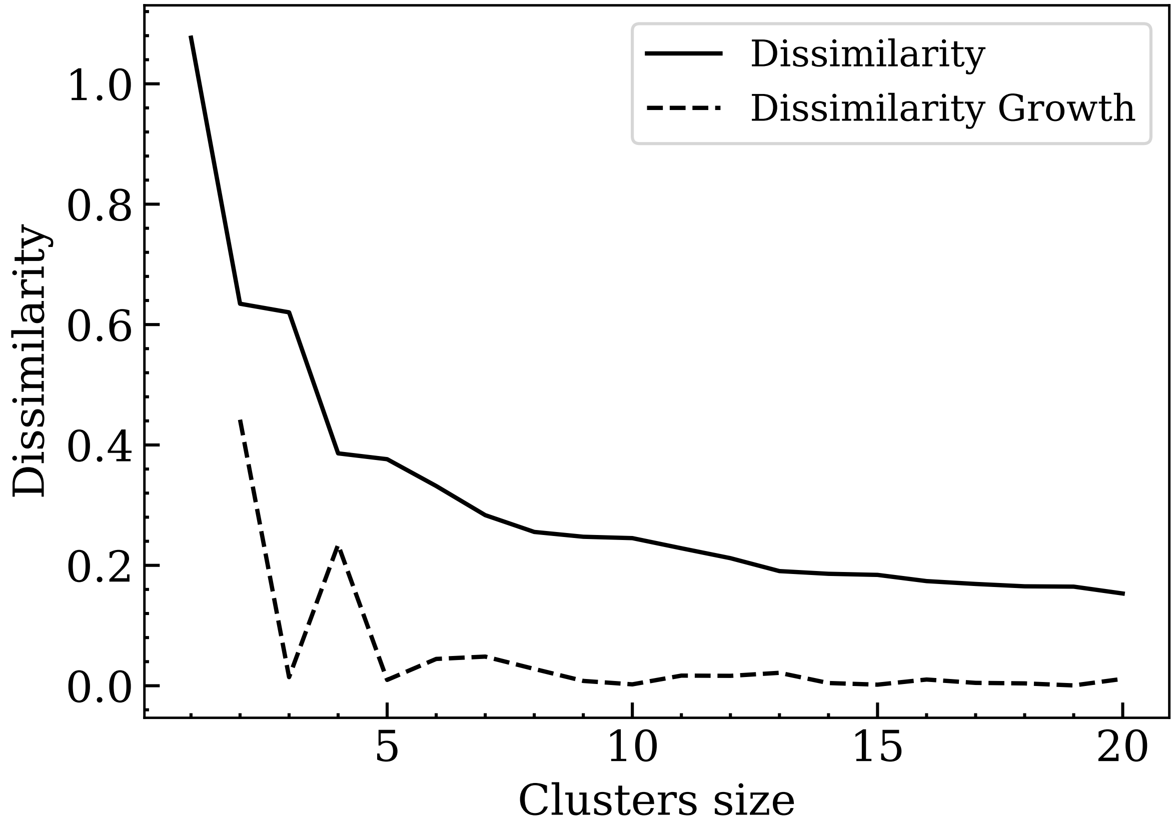

Fig. S1. The variations of dissimilarity (solid line) and dissimilarity growth (dashed line) in a function of cluster size, respectively. These metrics are used to select the optimal output cluster size, which is determined by an abrupt increase in dissimilarity growth (dashed line), resulting in four final clusters.

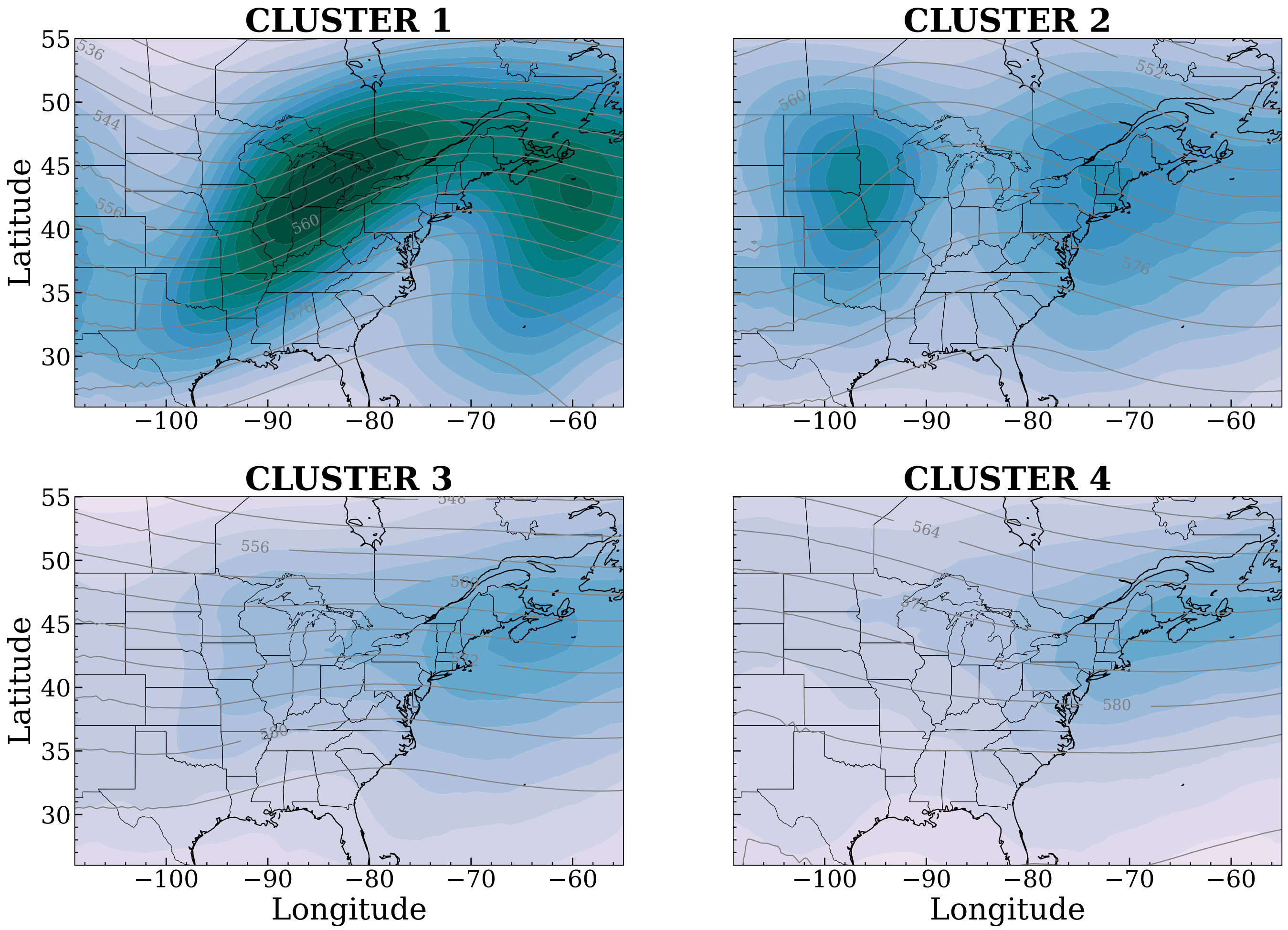

Fig. S2. The composite distributions of eddy kinetic energy (EKE) at 200 hPa (color fills; $m^2\ s^{-2}$) and Z500 (contours; m), for each cluster, respectively.

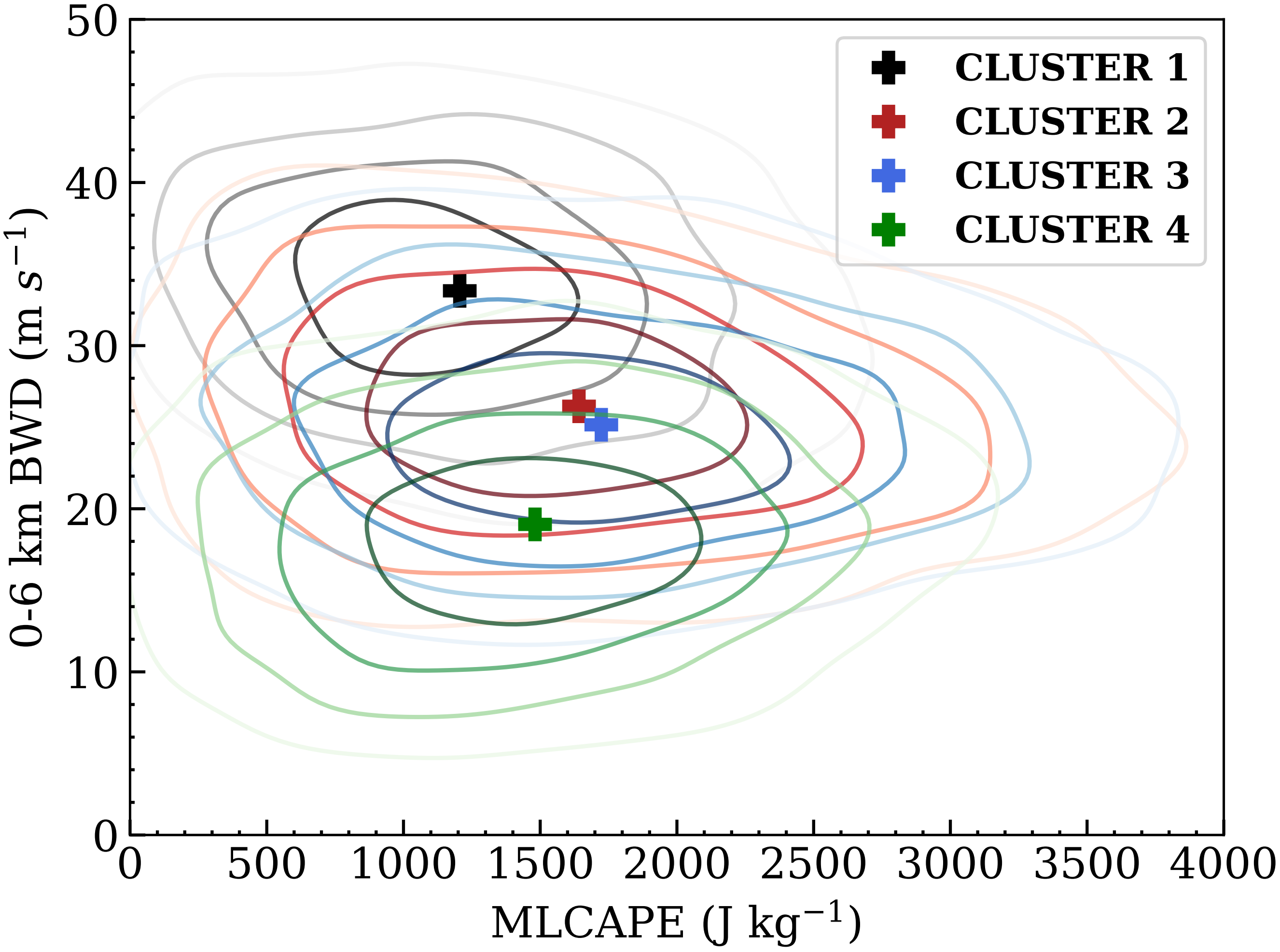

Fig. S3. The kernel density distributions of near-storm MLCAPE vs. BWD06 within each cluster, respectively. Each set of contours is centered on the region with the highest density of occurrences, with the innermost contour enclosing 15% of the data, and outward contours are in 15% intervals. The MLCAPE and BWD06 are selected as the maximum values in a 5°×5° area centered on the tornado report start location for each case. The plus signs denote the median values for each variable.

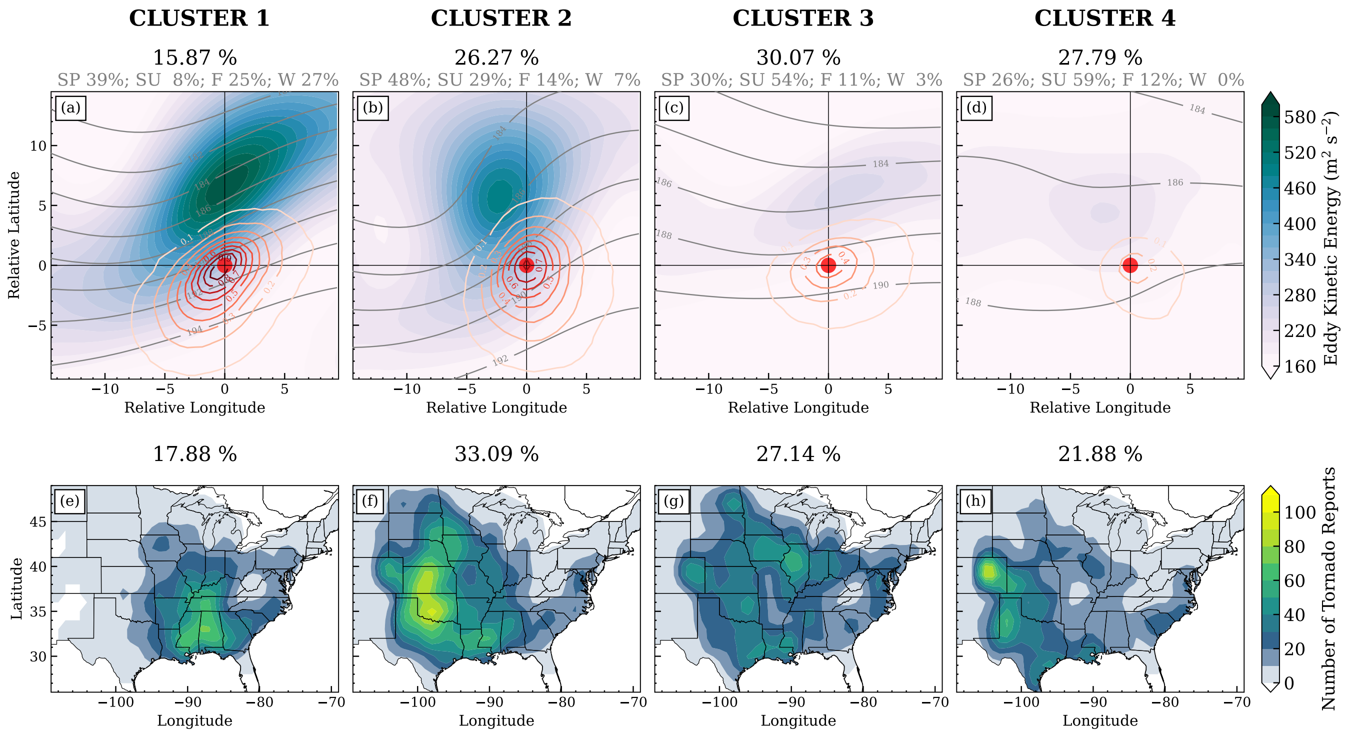

Fig. S4. As in Fig. 2 but using K-Mean clustering method.

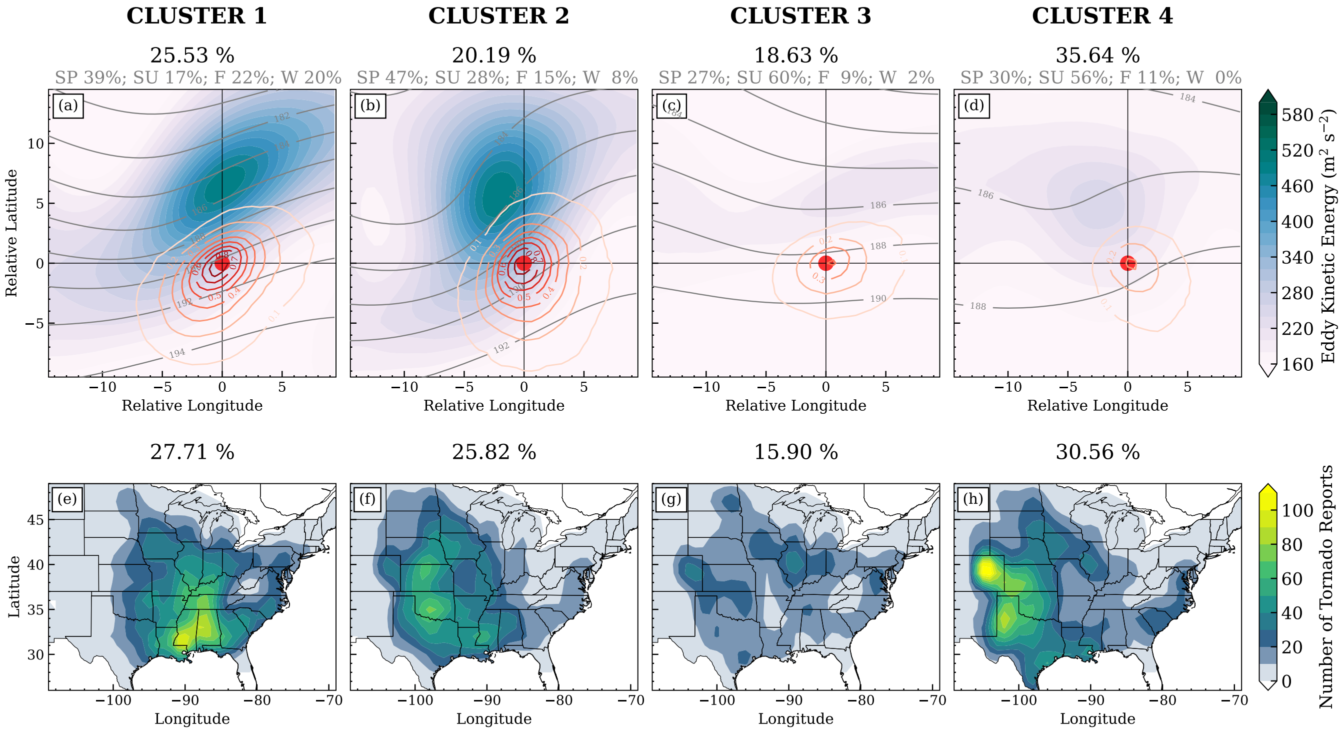

Fig. S5. As in Fig. 2 but using Self-Organization Map (SOM) clustering method. SOM is a neuron-network-based unsupervised machine learning algorithm that chooses samples at random at each iteration of training. Therefore, the results are also affected by sampling variability (e.g., different random seeds used in computation). The overall resulting synoptic patterns are similar to Fig. 2 in the main manuscript.

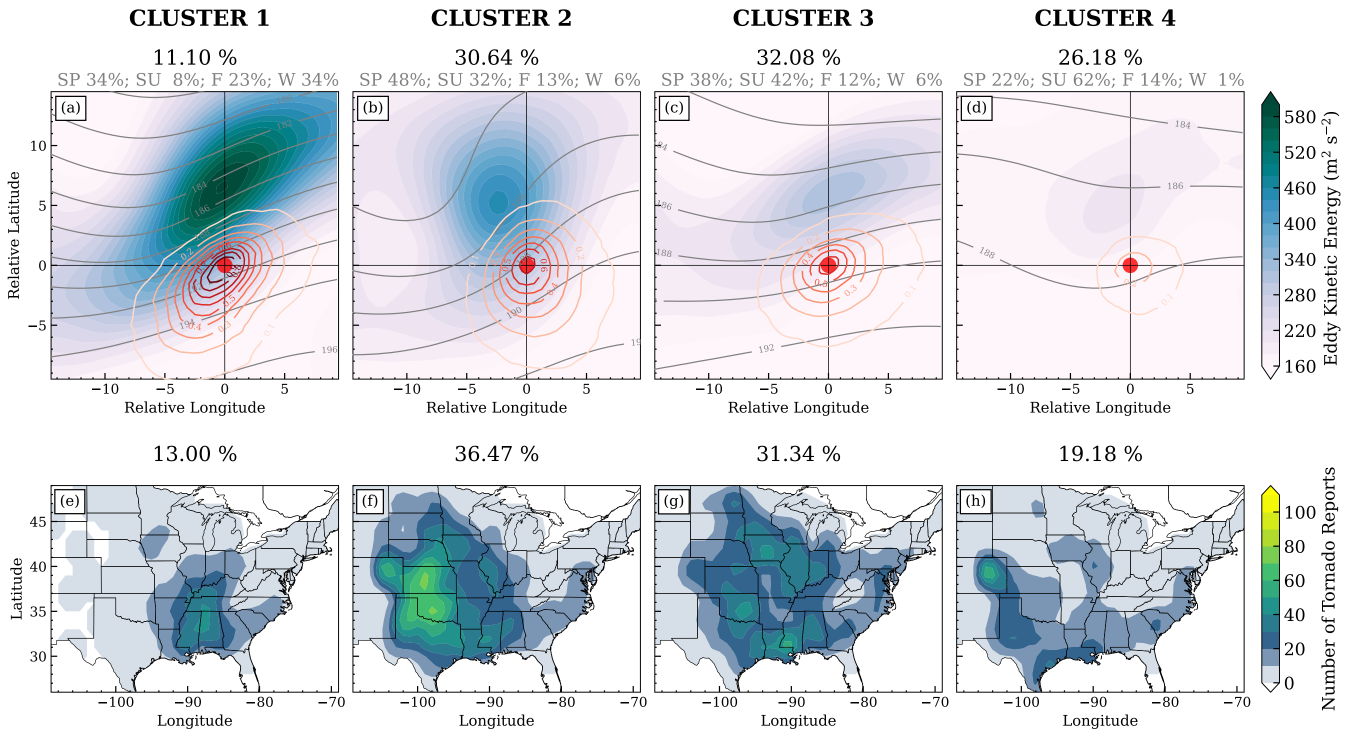

Fig. S6. As in Fig. 2 but randomly sampling 70% of EF0/EF1 tornadoes.

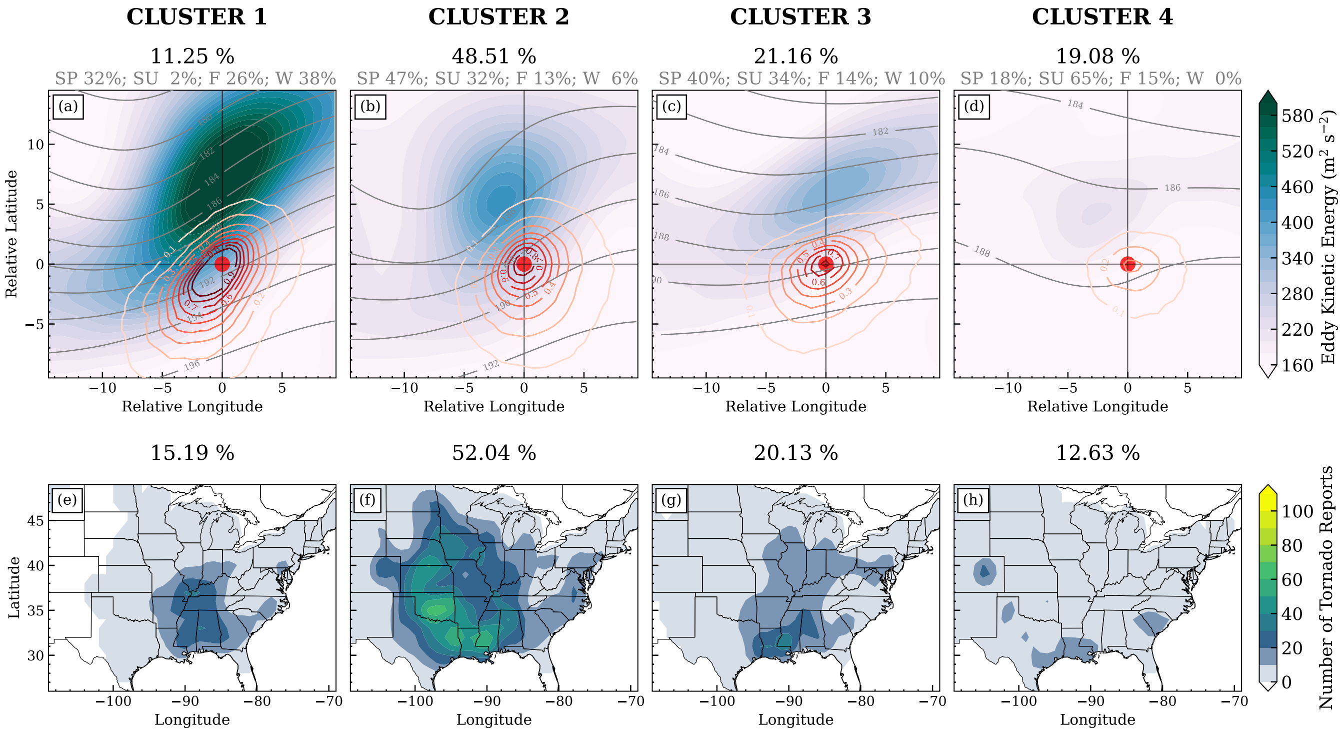

Fig. S7. As in Fig. 2 but using EF1 and greater (EF1+) tornado reports only. A total of 21153 EF1+ tornado reports are used associated with 8728 3-hourly weather maps.

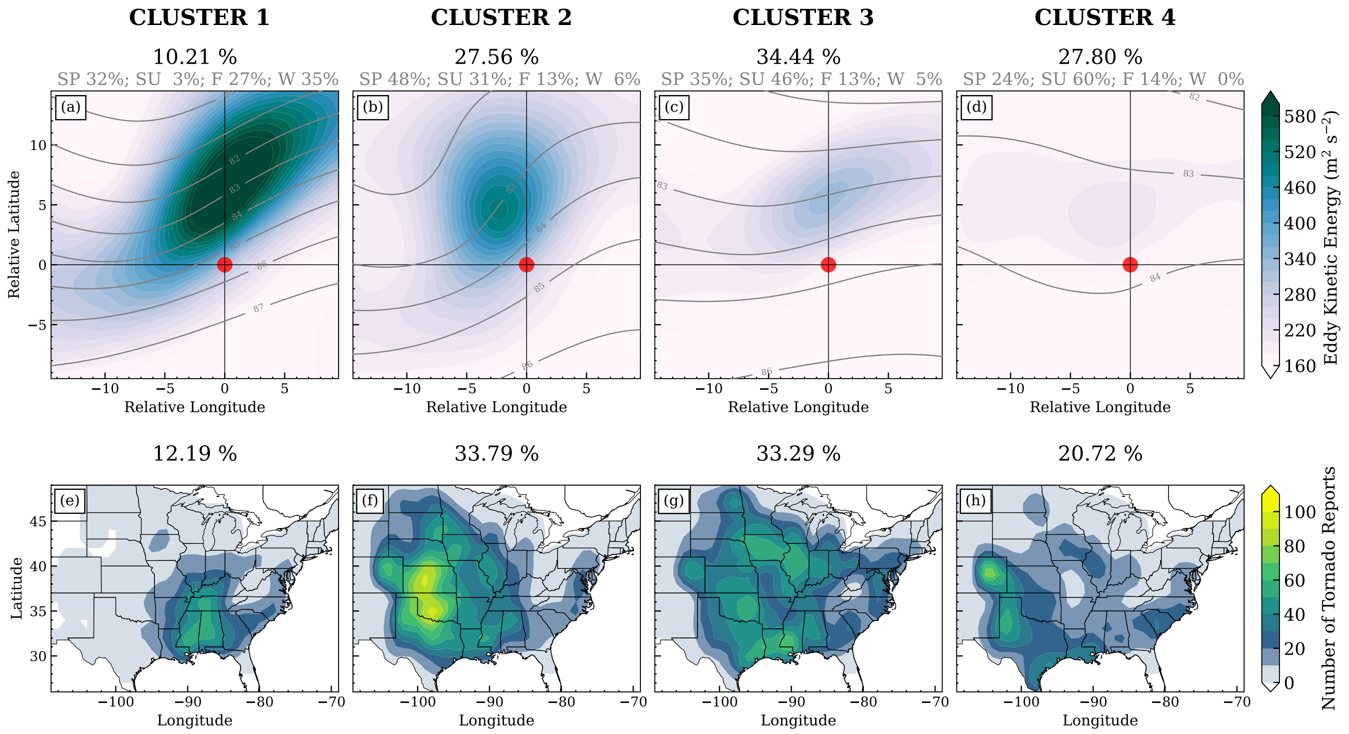

Fig. S8. As in Fig. 2 but using hourly ERA5 pressure level reanalysis dataset while maintaining their original resolution ($0.25^o \times 0.25^o$; no interpolation is performed). ERA5 has a higher temporal and spatial resolution than MERRA-2, resulting in more grid points within a fixed region and larger norms of the Z500 matrices. Therefore, the normalized Z500, shown as grey contours, is smaller than that in Fig. 2 in our manuscript; however, these differences are purely mathematical, and the internal distribution is what truly matters. The four resulting synoptic patterns are consistent with Fig. 2.

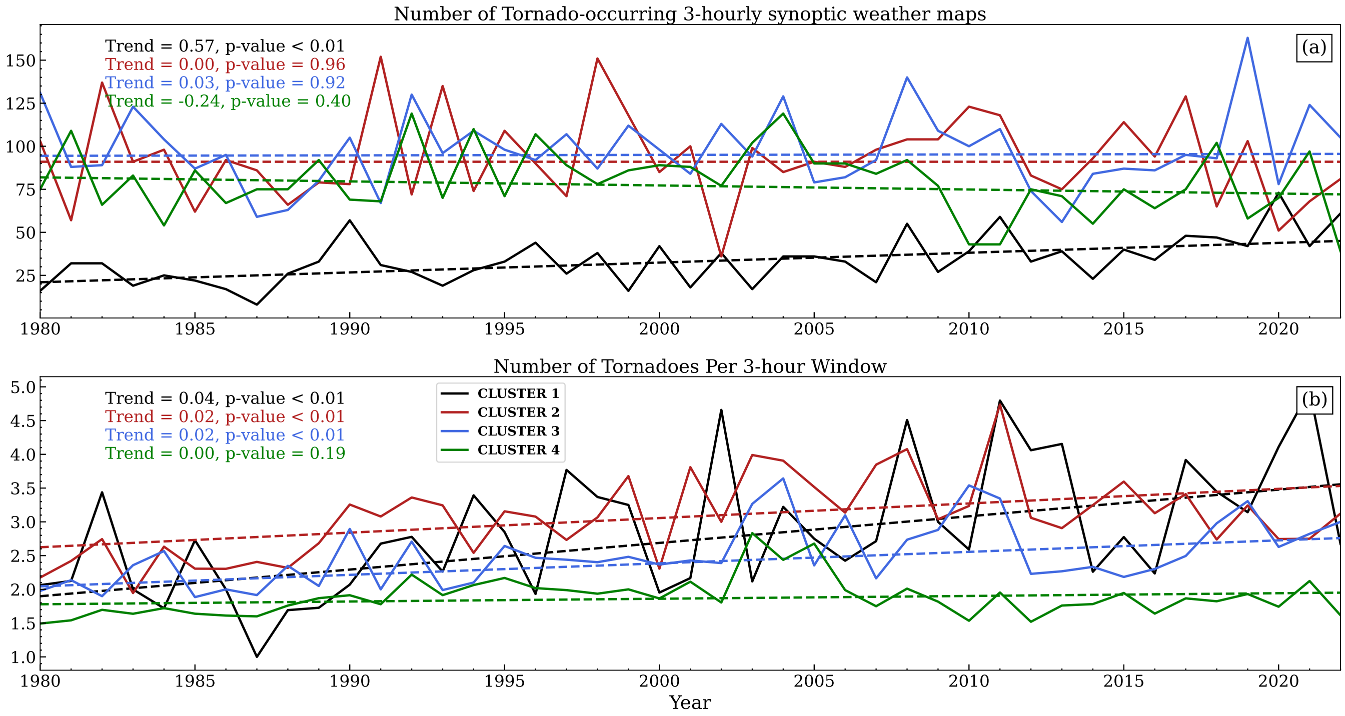

Fig. S9. As in Fig. 4 but randomly sampling 70% of EF0/EF1 tornadoes.

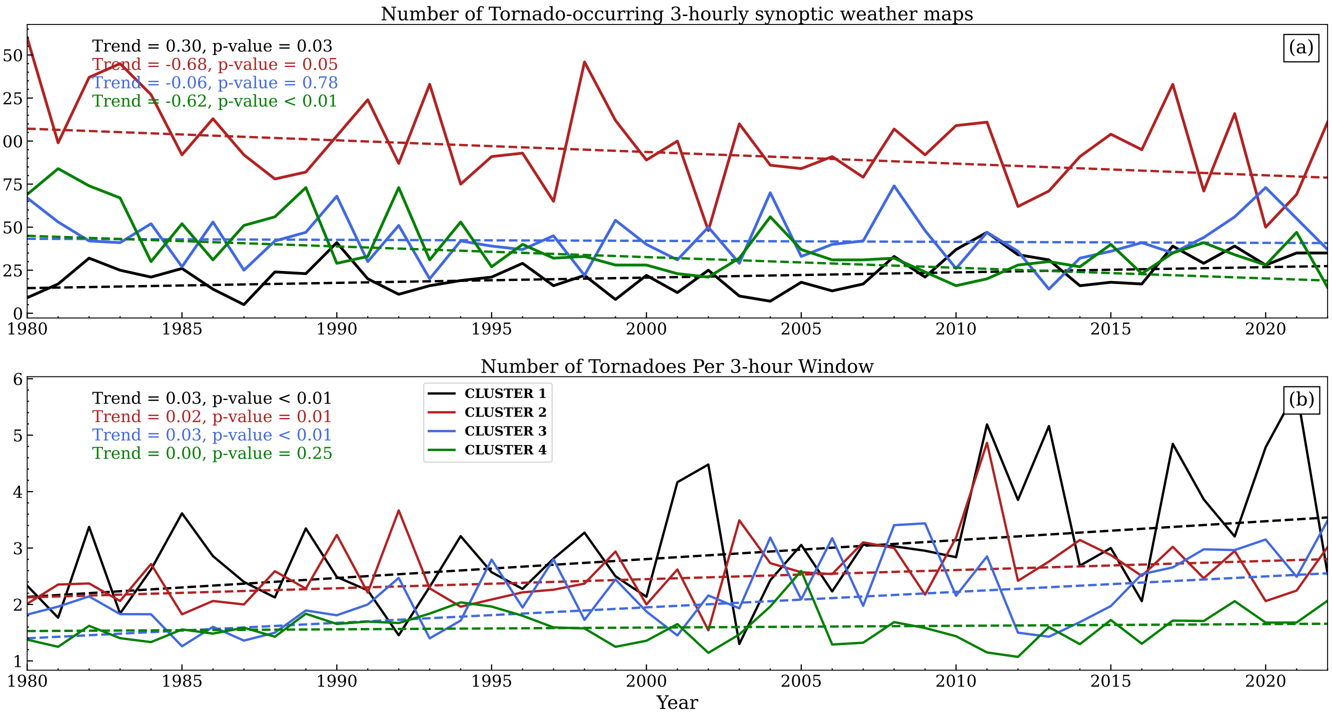

Fig. S10. As in Fig. 4 but using EF1 and greater (EF1+) tornado reports only.

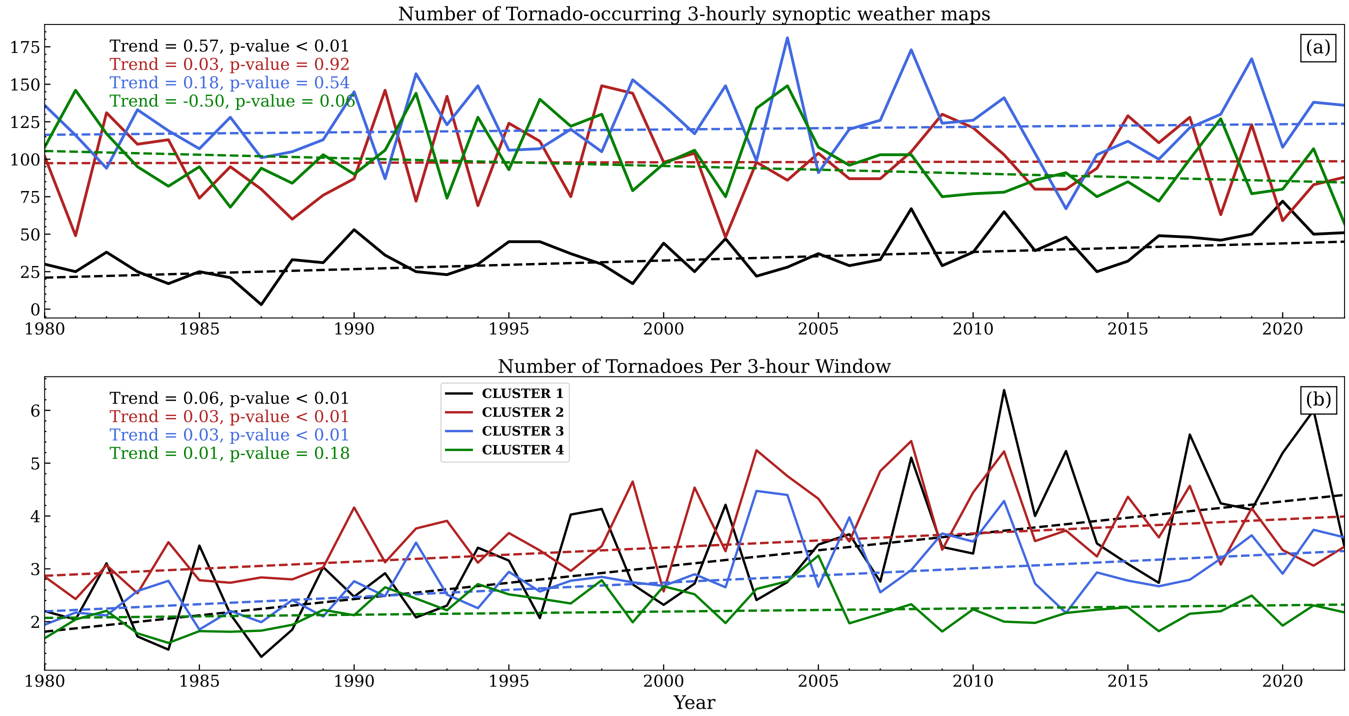

Fig. S11. As in Fig. 4 but using hourly ERA5 pressure level reanalysis dataset with their original resolutions.

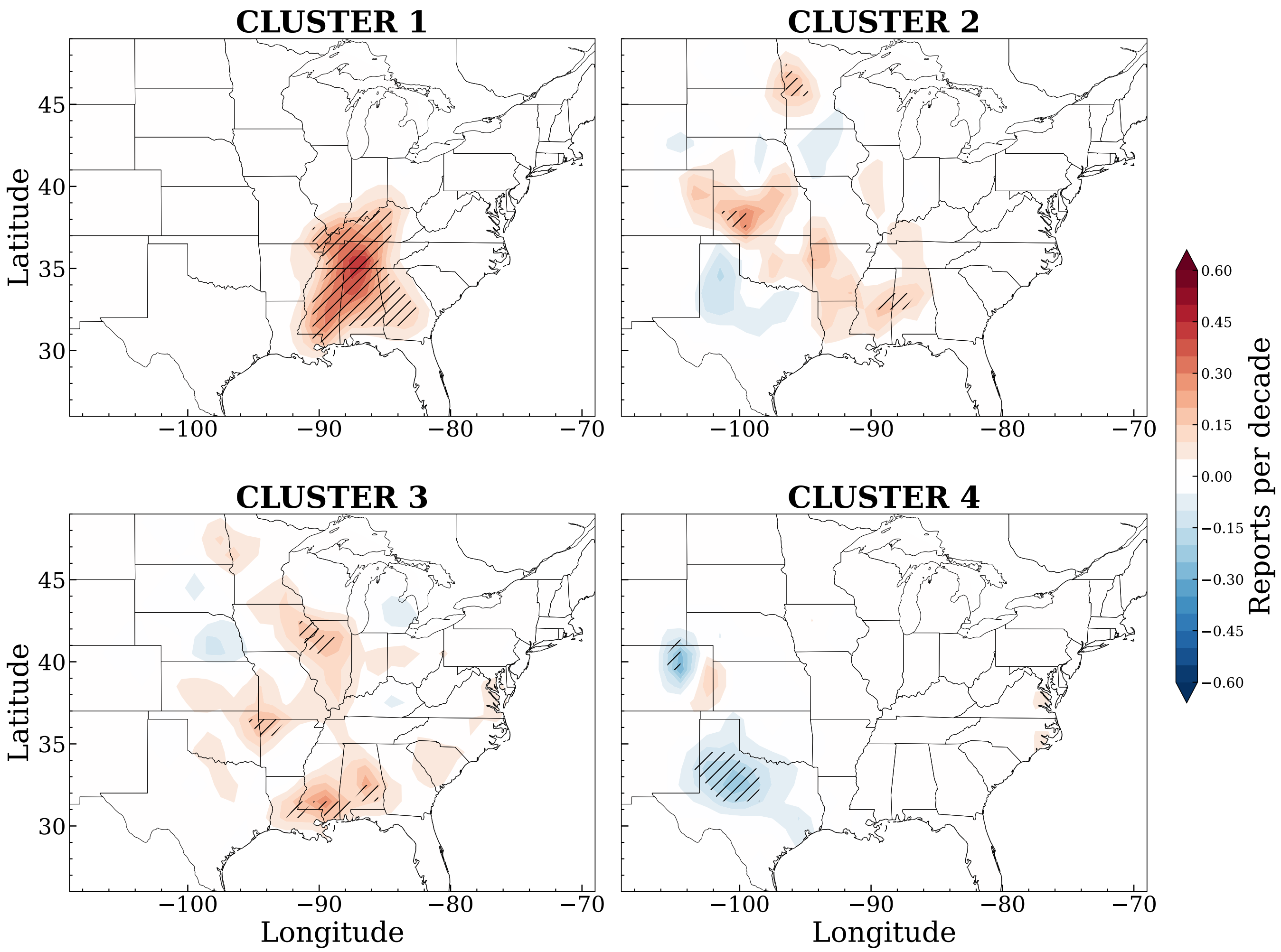

Fig. S12. As in Fig. 5 but randomly sampling 70% of EF0/EF1 tornadoes.

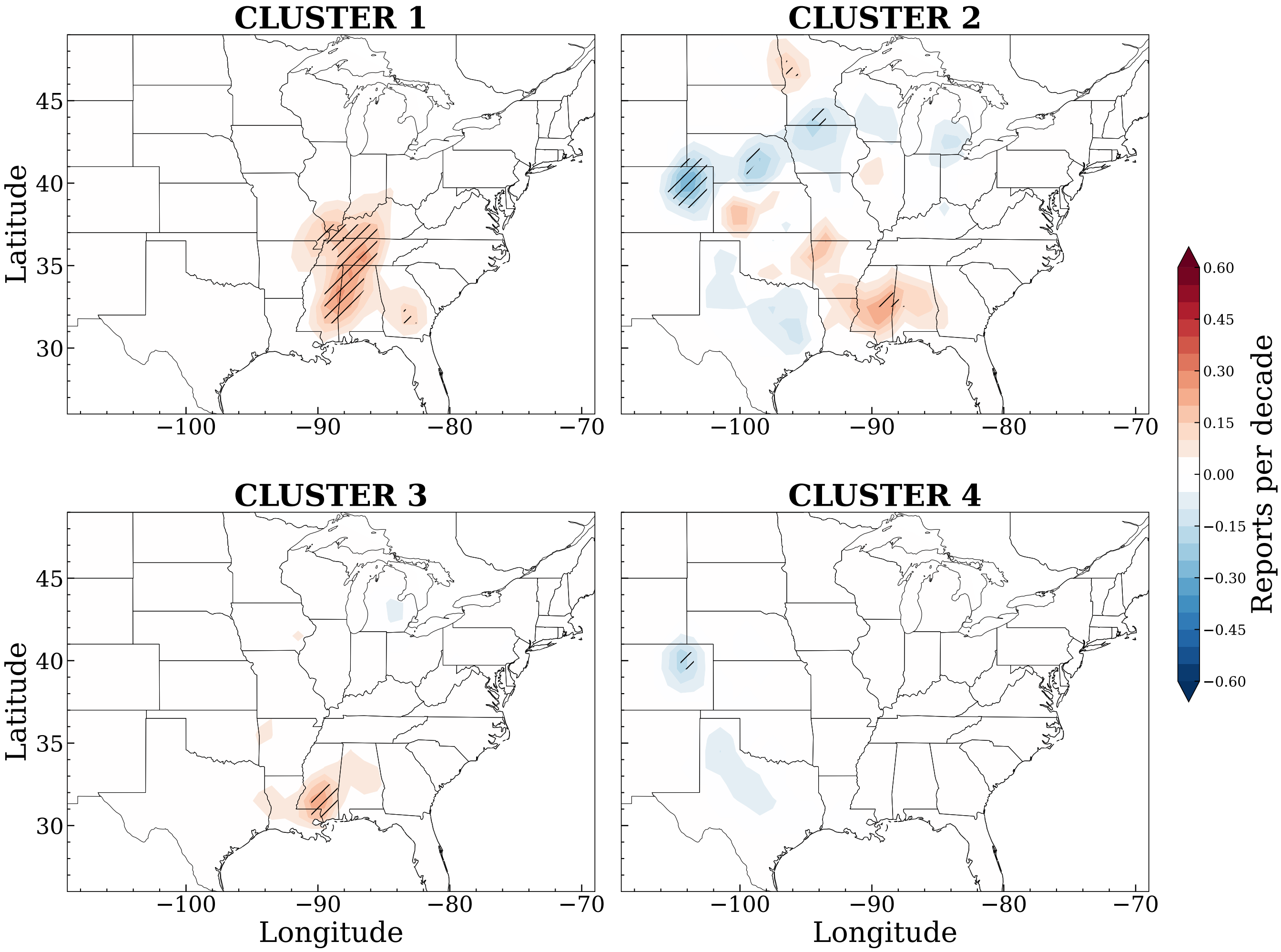

Fig. S13. As in Fig. 5 but using EF1 and greater (EF1+) tornado reports only. Cluster 1 continues to dominate the increasing trend in tornadoes in the southeast U.S. The main difference compared with Fig. 2 is that the decreasing trend in **Cluster 4** is less pronounced, which is expected, as the weaker synoptic forcing in **Cluster 4** is likely associated with weaker tornadoes, which were excluded from the analysis.

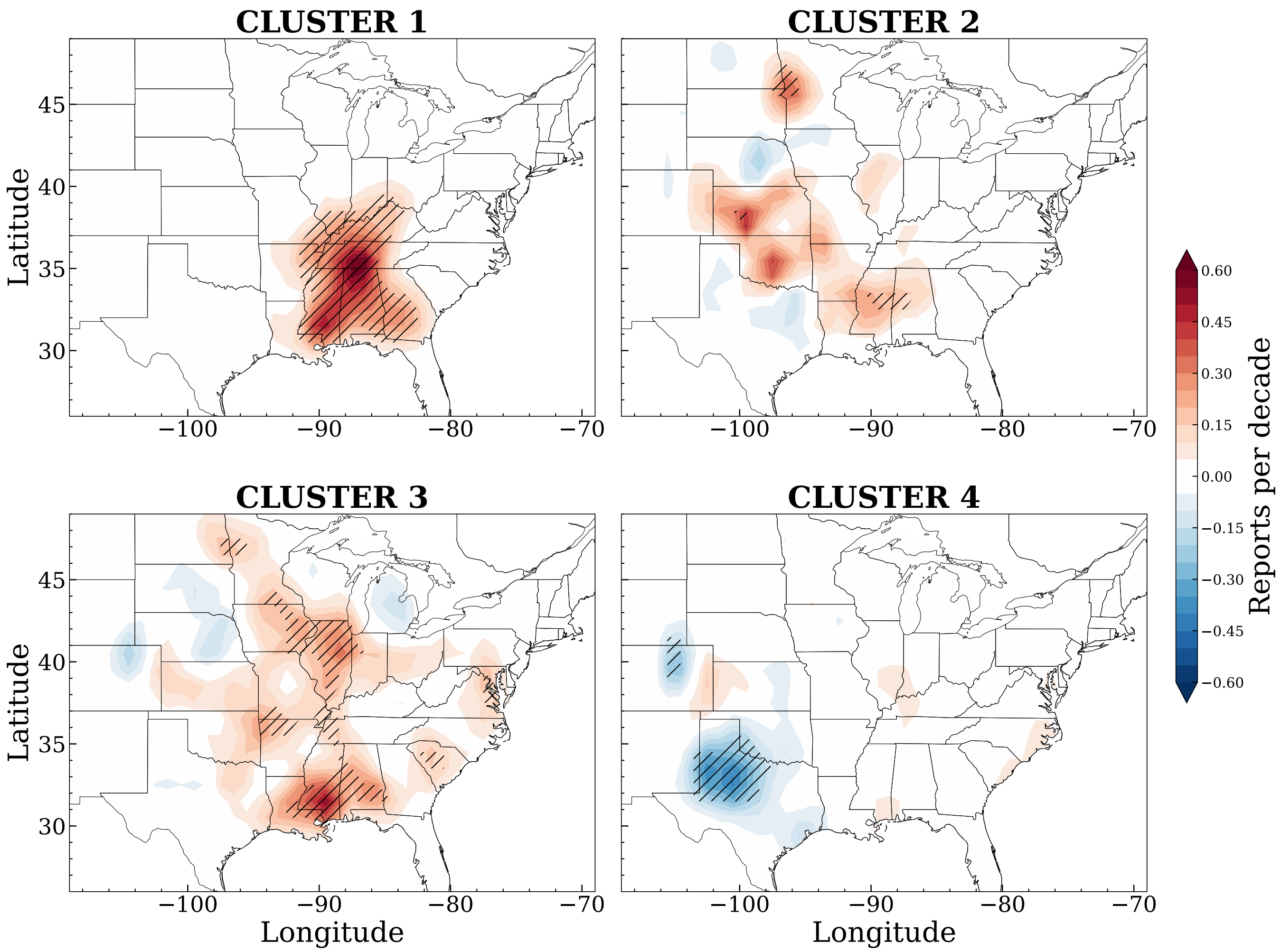

Fig. S14. As in Fig. 5 but using hourly ERA5 pressure level reanalysis dataset with their original resolutions.
